# Reliability and reproducibility of antinuclear antibody testing in pediatric rheumatology practice

**DOI:** 10.3389/fmed.2022.1071115

**Published:** 2023-01-09

**Authors:** Barbara E. Ostrov

**Affiliations:** Division of Pediatric Rheumatology, Department of Pediatrics, Bernard & Millie Duker Children's Hospital at Albany Med, Albany, NY, United States

**Keywords:** antinuclear antibodies, immunofluorescence assay, multiplex assay, pediatric rheumatology, reliability, reproducibility, Juvenile Idiopathic Arthritis, pediatric systemic lupus erythematosus

## Abstract

Antinuclear antibody (ANA) testing is common practice among health care practitioners when evaluating children and adolescents with non-specific symptoms including fatigue and aches and pains. When positive, ANA results often lead to referrals to pediatric rheumatologists as these antibodies may be key indicators for specific pediatric rheumatologic diagnoses. The reliability and reproducibility of ANA tests varies with assay techniques and validation and interpretation of results. In the following article, review of ANA testing in pediatrics is provided along with case examples that demonstrate the reliability and reproducibility of these results in specific scenarios common in the practice of pediatric rheumatology. Guidelines for more accurate utilization of ANA testing are presented with the aim to improve testing and interpretation by ordering clinicians.

## Introduction

Antinuclear antibodies (ANA) are among the most commonly ordered tests for patients referred to pediatric rheumatologists, with positive results leading to as many as 20% of rheumatology office referrals (1–3). Up to 70–90% of these children are not diagnosed with an inflammatory rheumatic disease at presentation. For the majority of these children, the symptoms for which testing had been done ultimately resolves without evolution to a rheumatic diagnosis (1, 2, 4, 5). Understandably, when informed of the possible clinical implications of a positive ANA, families and patients become very concerned. It is the role of the pediatric rheumatologist to inform and educate about possible explanations for this result, including that a positive ANA is present in many healthy children with no underlying diagnosis. Owing to this fact, the International Committee on Diagnostics (ICD) established a unique ICD-10 code (R76.0) for the specific diagnosis of “positive ANA” in order to bill for these consultations (6).

Determination of ANA results is crucial for diagnosis in some children and for determining prognosis in other children with specific rheumatic diseases. In the practice of pediatric rheumatology, upon consultation for a positive ANA, we may find results to be neither reliable in diagnosing a rheumatic disease nor reproducible from one assay technique to another. According to the dictionary, *reproducibility* indicates the extent to which consistent results are obtained when a test is repeated. *Reliability* indicates that a test result can be depended upon to be accurate. To maximize reliability and value of ANA testing, the American College of Rheumatology (ACR) together with the American Academy of Pediatrics (AAP), in their efforts as part of the “Choosing Wisely” campaign stated (7): “*Do not order ANA and other autoantibody testing on a child unless there is strong suspicion or specific signs of autoimmune disease*.” Regardless, the volume of referrals for a positive ANA to our practices remains high but does afford many opportunities for pediatric rheumatologists to educate our colleagues and families about ANA interpretation, reproducibility, and reliability.

In the following article, the utility of ANA determination in pediatric patients with possible rheumatic diseases is reviewed. Assay development is summarized, with particular attention paid to evidence-based data for using ANAs in children. The indications for accurate and timely diagnoses based on reliable and reproducible test results are reviewed. Specific recommendations and approaches to improve ANA ordering and interpretation in pediatric practice are provided.

## ANA testing and interpretation

ANA testing is considered part of the evaluation of patients with symptoms concerning for autoimmune disorders. This test is an important tool to diagnose pediatric systemic lupus erythematosus (pSLE) (4, 7) as a positive ANA result is a mandatory entry criterion in the most recent 2019 SLE diagnostic criteria (8). Other pediatric autoimmune diseases, including autoimmune hepatitis (AH), systemic scleroderma, and Mixed Connective Tissue Disease (MCTD), also include ANAs as part of their diagnostic criteria (see Table 1) (10, 11). In other diseases, ANAs are important in prognostication but may not be diagnostic. Examples include that ANA positivity is used to guide screening intervals by pediatric ophthalmologists for children with Juvenile Idiopathic Arthritis (JIA) at risk for uveitis (12), and that an elevated ANA suggests that a child with Raynaud's phenomena (RP) is more likely to develop a diagnosis such as MCTD or scleroderma (13, 14). In these clinical scenarios, ANA results must be reliable and reproducible in the laboratory or diagnoses may be missed or risk assessment delayed.

**Table 1:** Positive ANAs in pediatric patients [Adapted from (9)].

**Disease/Diagnosis**	**Percentage ANA +**
**Diseases in which** + **ANA is necessary in making diagnoses**	
• SLE • Systemic scleroderma	• 99–100% • 60–80%
**Diseases in which** + **ANA is helpful in making diagnoses**	
• Juvenile dermatomyositis • Primary Sjogren's syndrome	• 50–60% • 50%
**Diagnoses where** + **ANA is important in determining**	
**prognosis or informs monitoring protocol**	
• JIA–informs risk of developing uveitis and ophthalmologic screening protocol • Primary Raynaud's phenomena • Secondary Raynaud's due to an evolving disease, such as early scleroderma	• 60% • < 20% • 50–60%
**Diagnoses/diseases in which ANA** + **is part of diagnostic**	
**criteria**	
• SLE • Autoimmune hepatitis Type 1 • Autoimmune hepatitis Type 2 • Drug-induced lupus • Mixed Connective tissue disease	• 99–100% • 98–100% • 50% • 100% • 100%
**Scenarios in which ANA testing is not helpful for diagnosis or**	
**prognosis/monitoring but are commonly tested**	
• Rheumatoid arthritis • Fibromyalgia • Thyroid disease • Children in families with autoimmune disease who themselves have no signs or symptoms of autoimmunity	• 30–50% • 10–20% population norm for age • 30–70% • 5–30% population norm for age

Symptoms in children for which ANA tests are commonly ordered to evaluate for possible rheumatic diseases include persistent aches and pains, fatigue and/or possibly when parents/patients report unexplained fevers. Such symptoms lead referring practitioners to order testing due to concerns that their patient has an autoimmune disease. In their study aiming to understand the ordering practices and rationale for testing ANAs, Correll et al. identified that providers often ordered ANAs to screen for autoimmune disease in the setting of joint pain but with no inflammatory arthritis nor rash to suggest JIA or pSLE (4). The pre-test probability of a rheumatic diagnosis is low in children with non-specific joint pain, hence making the ANA a weak screening test. What confuses the situation further for the ordering provider is that ANAs are frequently positive in non-rheumatologic illnesses and in healthy children (15–18) (see Tables 2A, B). As many as 40% of children and teens report arthralgias at well-child visits (40), 30% report fatigue (41) and smaller numbers report unexplained fevers (18). The differential diagnosis for such symptoms is broad; many of these children have acute or chronic infections, non-rheumatologic autoimmune diagnoses, or other chronic illnesses (15, 18, 24, 25, 27, 30, 31). Uncommonly, an ANA may be identified during evaluation of a child who is ultimately diagnosed with a malignancy (28, 29). In actuality, finding a positive ANA in a child with a non-rheumatologic illness or who is otherwise healthy is more likely than identifying a rheumatologic explanation. This is further emphasized by noting the relatively low prevalence of pediatric rheumatic diseases being approximately 400/100,000 (0.4%) of children in the United States (3). Further, as the prevalence of positive ANA in healthy adolescents has increased to 30% over the past 30 years, it is increasingly likely that pediatric rheumatology referrals will be made for non-specific symptoms in the setting of a teen with a “positive ANA” (38).

**Table 2A:** Rheumatologic diagnoses with positive ANA.

**Rheumatologic diagnosis**	**% Positive**	**Pattern/Comments**	**References**
JIA			
- Oligoarticular - Polyarticular - Systemic	60% 30% < 5%	Homogeneous or speckled; low to moderate titer	(12, 19, 20)
SLE	99–100%	Homogeneous, peripheral or speckled Anti-ds-DNA and anti-Smith antibodies subserology	(8, 19)
Dermatomyositis	50–60%	Homogeneous Myositis-specific antibodies	(9)
Scleroderma	50–60%	Centromere pattern in Limited cutaneous scleroderma SCL-70 subserology in Diffuse cutaneous scleroderma	(21)
Sjogren's syndrome	50–60%	Homogeneous, nucleolar or speckled SS-A and SS-B antibody subserology	(9)
MCTD	100%	Speckled Positive RNP antibodies subserology	(11, 22)
Raynaud's phenomena			
-Primary - Secondary	< 10% 30–40%	Positive ANA portends risk of future CTD	(13, 14)
Vasculitis	Rare	Unless vasculitis presents as part of SLE or MCTD, for example, ANAs are not typically found	(11, 22, 23)

JIA, Juvenile idiopathic arthritis; SLE, systemic lupus erythematosus; ANCA, anti-neutrophil cytoplasmic antibodies; ANA, antinuclear antibodies; Anti-RNP, ribonucleoprotein antibodies; Anti-SS-A, SS-B, Sjogren's syndrome A, B antibodies; Anti-ds-DNA, double stranded DNA antibodies; SCL-70, scleroderma-70 antibodies; RP, Raynaud's phenomena; MCTD, mixed connective tissue disease.

**Table 2B:** Non-rheumatologic diagnoses with positive ANA.

**Non-rheumatologic diagnosis**	**% Positive**	**Comments**	**References**
Autoimmune thyroid disease	30–70%	Often subclinical thyroid disease with isolated + ANA	(24–26)
Autoimmune hepatitis Type 1 Autoimmune hepatitis Type 2	98–100% 50%	1/3 have no antibody other than + ANA Requires IFA test only	(10)
Celiac disease	25–30%	ENA found in 3–21%	(27)
Malignancies	17–30%	Varies	(28, 29)
Steatohepatitis	20–25%	Varies	(30)
Infectious diseases	Varies	Varies	(15, 18, 31, 32)
Drug-induced	Varies	8-fold increase with minocycline Common with anti-TNF agents	(33–37)
Healthy child	5–13% child 10–30% teen	Common in otherwise healthy individuals	(2, 16, 38, 39)

ANA, antinuclear antibodies; ENA, extractable nuclear antigens; IFA, immunofluorescence assay; TNF, tumor necrosis factor.

**Figure 1:**
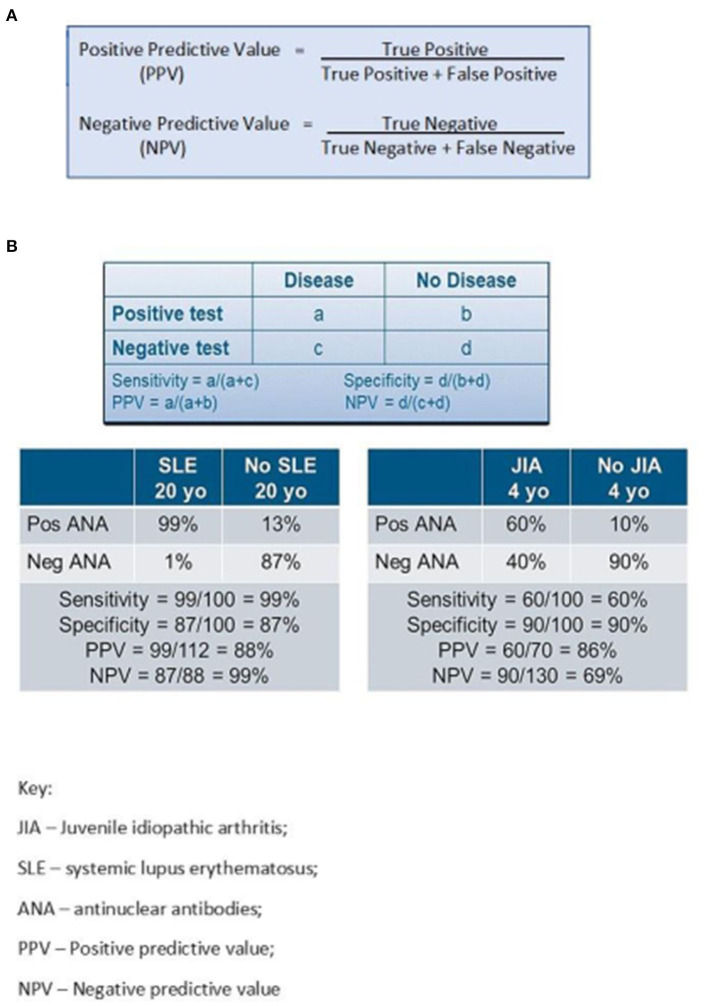
**(A, B)** Sensitivity, specificity, positive predictive value and negative predictive value of ANA testing.

The decision by a health care provider to order an ANA in clinical practice should ideally reflect an understanding of how the results will be used in clinical care. For any test result to be reliable and clinically useful, high pre-test probability should drive test ordering which is often not the case. Ideally a high “positive predictive value” (PPV) or “negative predictive value” (NPV) should help rule in or rule out a diagnosis, respectively. An ideal screening test would have 100% PPV and 100% NPV. However, the PPV and reliability of a positive ANA in specifying a diagnosis such as pSLE or identifying risk of complications such as uveitis in JIA is low (see Figure 1A). As the PPV of an ANA result used as a screening test in children is only 11% (9), the majority of patients with a positive ANA cannot be considered to have “screened positive” for a rheumatologic diagnosis.

In addition to PPV and NPV, one must understand the sensitivity and specificity of a test to use the results properly in patient care. Sensitivity measures true positive results, i.e., a person with SLE should have an elevated ANA titer (see Figure 1B). Sensitivity of reliable ANA testing is 97–99% indicating high probability that this test truly identifies SLE in a person who actually has this disease. On the other hand, specificity measures true negative results. A healthy person with no medical concerns should ideally have a negative ANA. This represents the probability that the test means the person does NOT have SLE when they are truly disease free. Since 5–13% of healthy children or up to 30% of healthy teens have a positive ANA not indicative of any identifiable diagnosis, the specificity of ANA in diagnosing SLE in adolescents is only 70%.

## ANA testing: History and methodology

The discovery of the LE-cell phenomenon in 1948 by Hargraves was early evidence of measurable immune response in patients with SLE and autoimmune diseases (42). The LE-cell forms due to phagocytosis of intact nuclei by mature polymorphonuclear leukocytes in bone marrow with phagocytosis by myeloid cells. In the 1950's and 1960's, antinuclear antibodies, anti-DNA antibodies, and antibodies to extractable nuclear antigens such as anti-Smith antibodies were identified [history reviewed in detail in (43)]. During these years, laboratory techniques were developed to better measure these antibodies: indirect immunofluorescence assay (IFA) and enzyme linked immunosorbent assay (ELISA).

The IFA technique initially used various cell lines as substrate but nowadays almost exclusively uses human epithelial type-2 cells (HEp-2 cells), the standard recommended by the ACR (44). The appropriately diluted patient serum is incubated with the substrate on the slide to allow autoantibodies to bind. The adherent antibody is then detected using a fluorescent labeled antisera containing anti-human IgG which can then be visualized by the microscope. The patient's serum is repeatedly diluted and reapplied to the substrate until no fluorescence is detectable; the dilution at which fluorescence was last visualized is the titer reported (9, 43). For children there is lack of consensus for the cut-off titer to consider a result to be positive; most laboratories use > 1:80 (45, 46). Some authors note that higher titers > 1:640 appear to be more specific for an evolving autoimmune disease (1, 39) but reports of high titers in non-rheumatic diseases abound (32, 33, 39, 47). The IFA technique also allows for visualization of fluorescence patterns: homogeneous is the most common and least specific; speckled, peripheral, nucleolar and centromere are less common patterns; centromere has the most specificity–for limited cutaneous scleroderma. A unique ANA pattern, dense fine speckled nuclear pattern (DFS, commonly referred to as DFS70), has been identified in adults largely without rheumatic or autoimmune diseases (48). DFS70 ANA patterns have been similarly analyzed in children finding low occurrence in about 2% of healthy children (49, 50) and 4–16% of children who also had a non-specifically positive ANA with other defined patterns (i.e., no associated autoimmune diagnosis) (49, 50). The presence of DSF70 pattern ANA by IFA has been identified in small numbers of patients with JIA-associated uveitis, localized scleroderma and juvenile dermatomyositis in some studies (49, 50). Based on other reports, however, it has been suggested that the presence of DSF70 antibodies along with an isolated positive ANA (i.e., no specific subserologies identified) more often indicates the lack of a chronic autoimmune disease (49, 50).

Laboratories are expected to summarize the titer as well as the pattern in their report to the ordering provider. Ideally ANA patterns would indicate specific diagnoses, but there is inconsistent pattern specificity for most rheumatic diseases (9, 51). ANA patterns are defined by an international consensus (51) and laboratories are expected to adhere to these guidelines. However, the IFA is a very manual procedure which requires repetitive dilutions and subjective interpretation of titers and pattern delineation by technicians with generally low volume throughput in the clinical laboratory.

In recent years, different assays including ELISA, fluorometric enzyme-linked immunoassay, and chemiluminescence immunoassay (CIA) have been added to the armamentarium of the clinical laboratory (9, 43) to advance ANA diagnostics further to more efficiently identify specific autoantibodies rather than just the ANA. Some of these assays are in a solid phase–a plate for ELISA or beads coated with relevant autoantigens in CIA. Patient serum is incubated with the solid phase components and autoantibodies to the specific antigens bind. In ELISA a detection antibody is linked to an enzyme that develops a colorimetric reaction. Similar methodology is used for dot or line blots with a visualizable colorimetric reaction. Techniques which add automation to immunofluorescence or colorimetric reaction facilitate reading a larger volume of samples in a rapid fashion thereby improving test throughput (52, 53).

**Figure 2:**
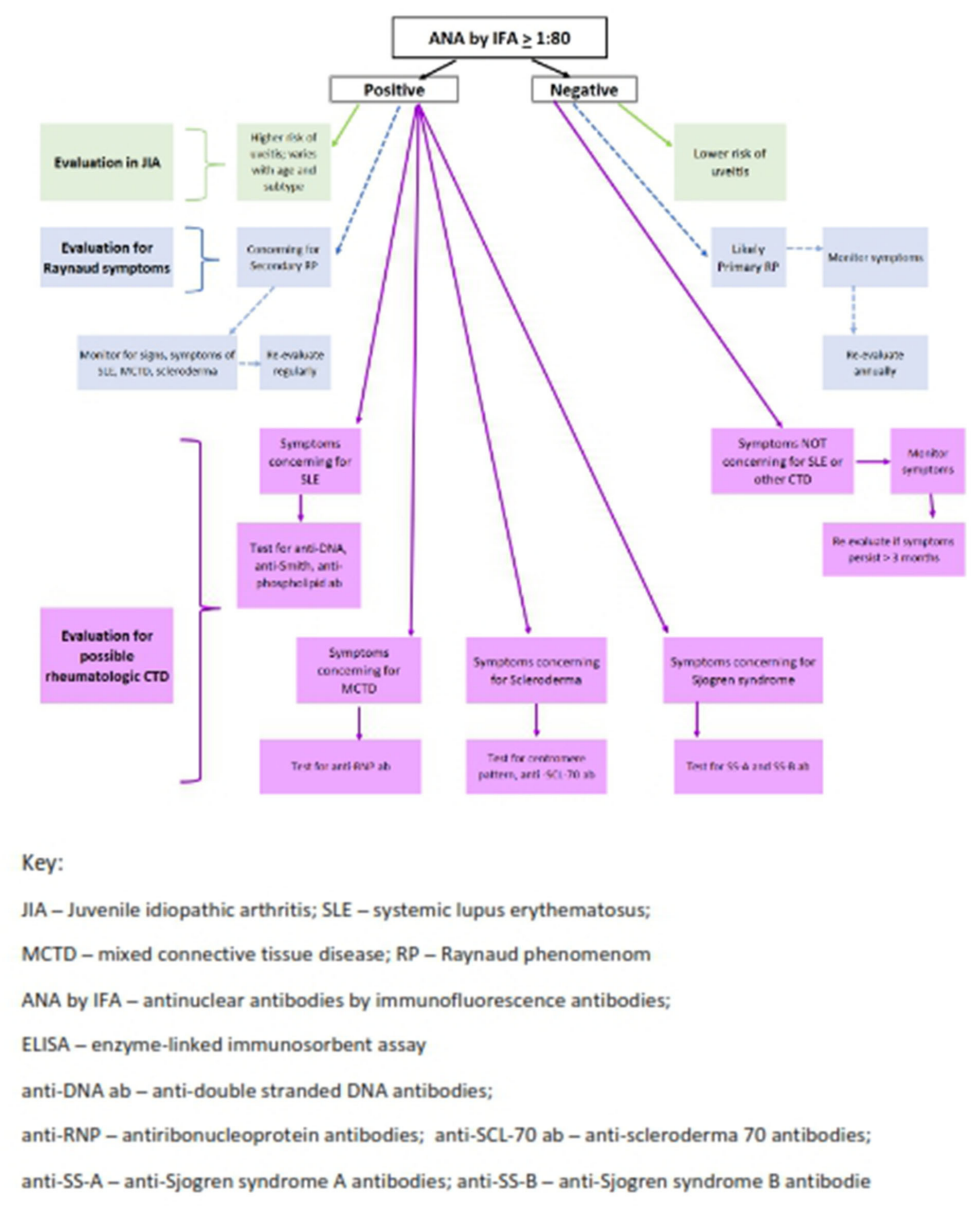
Algorithm for ANA testing in pediatric rheumatology. Two-step testing: ANA must be tested by IFA and subsequent antibodies can be tested by ELISA and/or Multiplex Assay.

Further efforts to improve efficiency and throughput in clinical laboratories have led to widespread use of semiquantitative solid-phase multiplex bead technology (45, 46). For these platforms, autoantigens are bound to a bead which has a distinct fluorescent signal. With this technique, simultaneous measurement of multiple autoantibodies that have adhered to the bead-containing autoantigens using either flow cytometry or LED technology (43) are measured. Multiplex assays can detect multiple autoantibodies relevant to specific rheumatic diseases at once; subserologies that are measured may include some of the following antibodies–or others–anti-double-stranded DNA antibodies, Sjogren Syndrome A (SS-A), Sjogren syndrome B (SS-B) antibodies, anti-Smith and anti-ribonucleoprotein (RNP), anti-SCL-70 antibodies, and centromere pattern ANA (Figure 2). A negative multiplex assay indicates the absence of the antibodies listed on the test package insert but may not actually indicate a negative ANA if specified antigens have not been identified nor included on the platform for the disorder in question (45, 54). It is important to recognize that in many rheumatic diseases, an ANA alone is the only positive serologic finding.

Xu et al. (46) confirmed that in pSLE multiplex assays were very good for detecting specific pre-selected antigens and were more efficient than ELISA or IFA, as multiple antibodies are tested at the same time improving throughput. These researchers identified strong correlation between multiplex results identifying specific subserologies using IFA or ELISA in pSLE, with 90% of samples demonstrating equivalent results (46). However, other authors found multiplex testing did not identify ANA results equivalent to IFA methodology in well-defined SLE sera (54). Somers et al. also identified discordant ANA results when comparing one assay type to another with weak agreement between IFA and multiplex assays in their study of a pediatric population (45). Multiplex assay sensitivity was only 22.2% (measuring true positivity; indicating low probability that this test truly identifies a person who actually has an ANA-related disease) and specificity was 92.7% (measures true negative). Additionally, in their study, 75% of sera with high titer IFA results were negative on the multiplex assay. Of similar concern, samples from children with arthritis demonstrated negative multiplex results in all 10 JIA study patients, each of whom had positive ANAs by IFA (46). Others documented that most patients with JIA-associated uveitis had negative ANAs by multiplex assays (19, 20), which is concerning as the screening protocol for chronic uveitis in JIA includes ANA positivity in young children with the oligoarticular subset (12). Sparchez et al. noted concordance between ELISA and IFA in pSLE but not in JIA (19). In AH, IFA is the recommended assay as more than 1/3 of patients do not have known antigens for use as part of multiplex platforms (10). These findings highlight the need to understand assay limitations when ordering and interpreting ANA test results.

Therefore, while the introduction of multiplex bead techniques seemed to be an innovative approach, in children with JIA and AH, there is low reliability and reproducibility. Few studies have been done in children to further assess intra-assay inconsistencies in other pediatric disorders. Melegari et al. (55) concluded that ANA by IFA should remain the “gold standard” as stated by the ACR (44).

Intra-laboratory variability in reporting ANA IFA titers and patterns has been a long-standing concern (52, 55, 56). Clinical laboratories vary in their ANA methodology and practice, test naming, and result report verbiage. Testing methodology and communication of results has not been standardized so often clinical laboratory reports may be misconstrued by primary care providers and even pediatric rheumatologists in their interpretation and hence approach to managing patients (56). ANA reports that list the result by stating “ANA Screen” without providing a titer is an important indicator that a multiplex assay of subserologies has been done rather than a titered ANA by IFA or ELISA (19, 46). This wording is confusing to many clinicians. These issues have led the ACR to assert that IFA using HEp-2 cells remain the “gold standard” assay. ACR recommendations also state that laboratories not using an ANA by IFA must specify assay methodology on their results report (44). The study by Naides et al. (56) documented that only 32–39% of clinical laboratories provided assay techniques in their ANA reports.

In order to more clearly demonstrate approaches to reliably and reproducibly use ANA tests, several real-world clinical examples are presented below. In each case, ANA testing was part of the patient evaluation that led to pediatric rheumatology referral. Each case exemplifies specific nuances related to ANA methodology and interpretation that have an impact on proper and timely diagnosis and treatment.

## Case examples

### Case 1: ANA in Juvenile Idiopathic Arthritis

A 2 year old presents with arthritis of her knee, wrist and ankle for 8 weeks. Her community primary care provider sent her blood tests to a commercial laboratory. Results summarized in consult request referral documents noted a normal complete blood count (CBC), an erythrocyte sedimentation rate (ESR) of 40 mm/h, a negative Rheumatoid factor (RF) and a negative ANA. She is referred to the pediatric rheumatologist for evaluation and treatment. Based on ACR criteria, the diagnosis of ANA negative oligoarticular JIA was made. Intra-articular glucocorticoids were administered. Given her negative ANA result, her risk of chronic anterior uveitis was considered moderate with recommended ophthalmologic examinations at baseline and every 6 months. However, at her initial visit with the pediatric ophthalmologist 6 weeks later, she was found to have moderate bilateral anterior uveitis; topical prednisolone drops were instituted. Upon further review, it was found that her initial ANA was performed at the commercial laboratory using multiplex solid-phase methodology. Upon repeating the ANA using the IFA technique, the result was positive in a titer of 1:320 in a homogeneous pattern consistent with that expected in a child with oligoarticular JIA and uveitis.

#### Concluding diagnosis

ANA positive oligoarticular JIA with high risk to develop chronic anterior uveitis. There was no sense of urgency initially to assess this patient for chronic anterior uveitis due to the false negative ANA result from unreliable multiplex test methodology which should not be used to assess uveitis risk in JIA.

#### Take home points

When evaluating a child for possible JIA, ANA testing is required for risk stratification regarding development of chronic anterior uveitis (Figure 2) (4, 57). ANA by IFA is the only reliable and reproducible technique in this scenario currently. Awareness of which assay the commercial laboratory employs is crucial to reliably interpret the results (56).

In Xu et al. all JIA patients had positive ANA by IFA but were negative on the multiplex assay (46). Since the multiplex panel includes only nuclear antigens never found in JIA, this methodology should never be used when assessing for risk of uveitis in this population (12). IFA titers nor patterns do not correlate with higher or lower risk of uveitis in oligoarticular JIA patients. While Schmerling et al. (49) identified higher proportion of positive DSF70 pattern ANAs by IFA in children with JIA and uveitis, these results have not been confirmed in subsequent reports (50). ANA titer levels have also not been clearly associated with risk of uveitis in JIA (9, 19, 45). Most concerning in cases like these is delayed or missed diagnosis due to the widespread use of multiplex solid phase immunoassays in many clinical laboratories and lack of awareness of this issue by most ordering providers. Overlooking the diagnosis of uveitis is potentially serious as this is an asymptomatic and vision threatening complication of JIA. Further analyses to study and improve these more cost-effective and efficient assays in children to improve reliability and reproducibility in JIA are needed.

### Case 2: ANA in pediatric systemic lupus erythematosus

A 14 year old has 1 month of fatigue with aches and pains, and white fingers with cold exposure. She is evaluated by her primary care physician and is noted to have blood pressure of 130/85, fever of 38° and a palatal ulcer. Musculoskeletal examination showed no weakness or swollen joints, but tenderness and pain with motion of wrists, knees and hips. Laboratory studies revealed leukopenia and lymphopenia with a white count of 3,000 cells/mm^3^ and lymphocytes of 400 cells/mm^3^. Anemia was present with a hemoglobin of 10.5 g/dl with normal indices. Platelets were slightly decreased at 110,000 cells/mm^3^. ESR was markedly elevated to 120 mm/h. Biochemical profile was normal with creatinine of 0.6 g/dl and normal liver function tests. “ANA Screen” was positive by multiplex immunoassay. Pediatric rheumatology consultant saw this teen 3 days later and confirmed a diagnosis of SLE with additional serologic testing: ANA by IFA positive in a titer of 1:1,280 peripheral pattern; elevated anti-double stranded DNA, anti-Smith and anti-SS-A antibodies. Other serologies were negative. Complement C3 was low at 45 g/dl (normal 90–130 g/dl) and C4 was unmeasurable at 0 (normal 25–50 g/dl). Urinalysis was clear. Hydroxychloroquine and low dose prednisone were instituted. One month later, the patient developed proteinuria and hematuria leading to a kidney biopsy with a diagnosis of diffuse proliferative glomerulonephritis.

#### Concluding diagnosis

pSLE with positive ANA Screen by multiplex assay further confirmed with IFA and titer, along with specific subserologies.

#### Take home points

In SLE, the multiplex assay often aligns with IFA and ELISA results (19, 46) however a detailed serologic picture beyond ANA determination is needed to understand the likely disease course in SLE. For example, high double stranded DNA (anti-ds-DNA) antibodies in the setting of low complement levels are often indicative of impending nephritis; anti-SS-A antibodies usually indicate risk of rash and photosensitivity (see Figure 2). In addition, rising titers of anti-ds-DNA antibodies as well as dropping levels of complement C3 and C4 correlate with increased disease activity and can be used to monitor SLE patients (58). ANAs and other subserologies do not consistently fluctuate with disease activity.

It is possible that ordering primary care providers are uncertain how to interpret “ANA Screen positive” results. Given the clinical presentation, this patient was diagnosed in a timely fashion. Obtaining the results of the entire multiplex subserology panel would have quickly indicated the patient's disease profile even earlier in her course. Monitoring the anti-ds-DNA titers by multiplex assays can be performed over time to assess and monitor such patients. ANA titers do not correlate with disease activity in a similar manner, hence there is no utility to repeat the ANA testing in such scenarios (58).

ANA patterns are not specific for diagnosing SLE although the peripheral or rim pattern is often found when anti-ds- DNA antibodies are present (51). The preponderance of pSLE patients have homogeneous patterns, with speckled, peripheral, centromere and two or more patterns reported less often (21, 51). The lack of specificity of IFA patterns, except for centromere in scleroderma or primary biliary cirrhosis, tells us not to rely on them for any diagnostic or prognostic guidance (51).

A positive ANA has been part of diagnostic criteria for SLE since the 1970's. The most recent ACR/EULAR criteria require positive ANA (at least 1:80 titer by IFA) as an obligatory entry criterion in order to make this diagnosis. The new criteria have sensitivity of 96.1% and specificity of 93.4% in adults (8). However, in pSLE these new criteria do not perform as well: 88.5% specificity as compared to the 1997 ACR set with 94.8% specificity, possibly due to weighing features in adults differently than in pSLE, such as more pronounced arthritis and hematologic manifestations in children (23).

This patient also had symptoms to suggest Raynaud's phenomena (RP) which may be a presenting sign of pSLE as well as other pediatric autoimmune diseases including scleroderma and MCTD. Fifty-percent of young children and 80% of adolescent patients with RP have no underlying autoimmune disease, i.e., primary RP (13). Multiplex assays have excellent reliability and reproducibility for identifying antibodies to nuclear antigens associated with systemic and limited scleroderma in some patients, but in others solely with RP without known nuclear antigens, measurement of the ANA by IFA is required (21) as the multiplex assay will be falsely negative. Centromere pattern ANAs suggest possible limited scleroderma and SCL70 subserology positivity suggests diffuse cutaneous scleroderma, with each serologic finding indicating the need to consider these etiologies in a child with RP.

A positive ANA provides 67% sensitivity and 41% specificity for the diagnosis of secondary RP (57). ANA positivity in children with RP is a key indicator of subclinical autoimmune disease that may evolve over time (14). Clinical history and examination with assessment of nailfold capillary microscopy plus ANA by IFA followed by monitoring over several years is currently the recommended approach to children with isolated RP (13, 14).

A high titer positive ANA is also part of the diagnostic criteria for MCTD (11). This overlap syndrome has combined features of SLE, scleroderma and inflammatory myositis. Inflammatory polyarthritis, RP and interstitial lung disease later in the course are typical features. ANA titers are generally very high, > 1:1,000 titer, and usually in a speckled pattern (11). ANA and anti-RNP antibodies are positive in 100% of children with MCTD (see Figure 2) (22, 59). Hetlevik and colleagues identified that high titer anti-RNP antibodies remained positive and were predictive of on-going disease activity in long-standing MCTD patients (22). RNP is often an included antigen on multiplex platforms so should be highly reliable and reproducible in diagnosing MCTD patients (59).

### Case 3: Drug induced ANA in pediatrics

A 16 year old is healthy except for moderate acne. He failed topical therapies and was placed on minocycline. Nine months later, he began to experience joint and muscle pain, intermittent fevers, mild facial rash. Laboratory studies revealed leukopenia with a white cell count of 2,500 cells/mm^3^ and lymphopenia with absolute lymphocyte count of 500 cells/mm^3^. He was anemic with hemoglobin of 10.5 g/dl but normal indices and had a normal platelet count of 240,000 cells/mm^3^. ESR was 50 mm/h, aspartate transaminase (AST) and alanine transaminase (ALT) were both elevated to 50 mg/dl and 65 mg/dl, respectively. The ANA by multiplex assay was negative. A diagnosis of SLE was considered by his referring physician who had expected the ANA to be positive. Upon pediatric rheumatology consultation, minocycline-induced autoimmune disease was considered most likely. Additional laboratory studies revealed the ANA by IFA was positive in a titer of 1:640 in a homogeneous pattern and also revealed antineutrophil cytoplasmic antibodies (ANCA) elevated in a titer of 1:1,280 in a perinuclear pattern; all other lupus and vasculitis serologies were negative. Anti-histone antibodies and anti-smooth muscle antibodies were also negative. Minocycline was thought to be the trigger for the autoimmune syndrome with some features of SLE and others to suggest possible AH. Minocycline was held and symptomatic treatment was given. Within 3 months all symptoms resolved. One year later the ANA was negative and the ANCA had fallen to a low titer of 1:80 in a perinuclear pattern. CBC, ESR and liver function tests were all normal.

#### Concluding diagnosis

Drug-induced lupus (DIL) and AH due to minocycline with positive ANA by IFA and ANCA by IFA.

#### Take home points

Drug-induced ANAs are common with some medications and should be considered when evaluating an unexplained ANA result. As an example, while not commonly prescribed for children, procainamide leads to a positive ANA in most patients within 12 months of use with no signs or symptoms of illness. In a subset of these drug-induced ANA patients, DIL may appear after up to 4 years of drug use (34). This is thought to be due to slow acetylator status (34). Anti-histone antibodies also develop in some patients. In pediatrics, minocycline has become one of the most common triggers of drug-induced autoimmunity, with up to an 8-fold risk with chronic use for acne in some reports (35). In DIL, the symptoms are somewhat different than idiopathic pSLE: arthralgia, myalgia, fever, malaise, and serositis are typical, but renal or neurologic lupus are rare. Laboratory findings include cytopenias as seen in this case. Anti-histone antibodies are found in classic procainamide and hydralazine DIL but rarely identified with minocycline. In addition, the presence of ANCA with autoimmune liver involvement is typical for DIL due to minocycline and propylthiouracil but not with other agents. When evaluating a patient for possible drug-induced autoimmune syndrome, be aware of the potential for a mixed autoantibody profile as found in this case. The negative multiplex assay in this case exemplifies the point that with the lack of specified nuclear antigens on a multiplex assay platform, ANA by IFA is the preferred method for reliable antibody determination.

Treatment for DIL primarily involves discontinuation of the medication and supportive therapy. Most children will remit within 3 months. Hematologic and hepatic laboratory changes may disappear over several months, but serologic changes may persist for 6 months or longer (34, 35).

Biologic agents and monoclonal antibody therapies have been increasingly prescribed for autoimmune and inflammatory diseases over the past 20 years (37). DIL attributed to biologic agents can be challenging to diagnose given the child's pre-existing autoimmune disorder for which the biologic agent is prescribed. Studies on infliximab in patients with inflammatory bowel disease have demonstrated development of ANAs and anti-double stranded DNA antibodies over time, however few patients develop DIL syndromes (33, 36, 37). Other anti-TNF agents including adalimumab and etanercept, are reportedly able to trigger autoantibodies as well as rare drug-induced autoimmune illness (36). Medication discontinuation is the approach used although this can be challenging depending on the underlying disease activity.

### Case 4: ANA in non-rheumatologic pediatric autoimmune disease

A 13 year old was referred to pediatric rheumatology because of fatigue, knee pain and a positive ANA. She had been experiencing knee pain for the past year and had been diagnosed with patellofemoral syndrome. Nonsteroidal anti-inflammatory drugs (NSAID) and physical therapy were not helpful. She reported no joint swelling or stiffness but only knee pain with climbing stairs and after arising from a seated position. Laboratory studies revealed a normal CBC and ESR, and an ANA by IFA of 1:80 in a speckled pattern. On exam, the patient was noted to have short stature with height at the 1^st^ %ile and weight at the 5^th^ %ile. Her thyroid was palpable but not tender. Musculoskeletal exam revealed no inflammatory arthritis; she had a positive grind test of both knees with full range of motion. Subsequent laboratory studies revealed a TSH > 100 mIU/L (normal 2–5 mIU/L) and free T4 that was 0.5 ug/dl (normal 5–8 ug/dl) anti-thyroid microsomal antibodies strongly positive at 100 IU/ml (normal is < 9 IU/ml). This teen was diagnosed with severe hypothyroidism and autoimmune thyroiditis which was the cause of her positive ANA.

#### Concluding diagnosis

Hypothyroidism due to Hashimoto's thyroiditis with positive ANA by IFA.

#### Take home points

Positive ANA is common in thyroid disease, with 30–70% of patients with autoimmune thyroid disease having a positive ANA by IFA (24, 25). Torok et al. reported elevated ANA by IFA in 30% of children with high titer thyroid antibodies in Hashimoto's thyroiditis (chronic lymphocytic thyroiditis), anti-thyroid globulin and anti-thyroid peroxidase antibodies as compared to the general pediatric population (24). There was no correlation of titer or pattern in predicting the existence of thyroid antibodies. One must always consider subclinical thyroiditis in children with non-specific rheumatic symptoms and a positive ANA (25, 26). With the frequency of thyroid antibodies indicative of impending thyroiditis in ANA positive children and non-specific symptoms, it is suggested that evaluation of anti-thyroglobulin and anti-thyroid peroxidase antibodies along with TSH and Free T4 be considered in the work up of unexplained ANA-positive children.

Another common ANA positive autoimmune disease not typically managed by rheumatologists is celiac disease, gluten enteropathy. While diarrhea, growth concerns and anemia are classic presentations in children, teens and adults can present with joint pain, fatigue and non-specific symptoms leading to consideration of rheumatic diseases. About 24–30% of celiac patients are ANA positive by IFA with some of these children also demonstrating nuclear antigens on further testing (27). The presence of ANA positivity may also signal association of celiac disease with other autoimmune diseases such as Sjogren's syndrome and SLE.

AH can also present to pediatric rheumatologists due to non-specific aches and pains and fatigue with a positive ANA. Laboratory studies may show cytopenias and elevated liver function tests. Nearly 100% of type 1 AH is ANA positive by IFA (10). Typical subserologies in AH include anti-smooth muscle and anti-(liver-kidney-microsomal) LKM antibodies. These may be included in some multiplex panels but since specific nuclear antigens are unknown in more than 1/3 of AH cases, multiplex assays may lead to false negative ANA results. Missing the diagnosis of AH might have severe clinical consequences due to delayed diagnosis and treatment of chronic liver disease. It is recommended that only ANA using IFA on HEp2 cells be used to evaluate AH.

### Case 5: Virally induced ANA in pediatrics

A 17 year old presents with a macular facial and body rash, low grade fevers, fatigue and joint pain with swelling and stiffness of multiple hand joints for 2 weeks. His 20 year old sister has SLE and the patient is concerned about this possibility. His laboratory tests reveal leukopenia and neutropenia with a white count of 2,000 cells/mm^3^, neutrophils 400 cells/mm^3^ and lymphocytes of 1,500 cells/mm^3^. Anemia was present with a hemoglobin of 8.5 g/dl with normal indices. Platelets were decreased at 80,000 cells/mm^3^. ANA was negative by an ANA multiplex assay. ESR was mildly elevated to 40 mm/h. Biochemical profile: normal creatinine of 0.6 g/dl and elevated ALT and AST of 80 mg/dl for each. Pediatric rheumatology consultant ordered additional testing including ANA by IFA which was positive in a titer of 1:640; other subserologies were negative including anti-double stranded DNA, anti-Smith and anticardiolipin antibodies. Complement C3 and C4 were normal and urinalysis was clear. Given the brief course, concern for viral syndrome led to Parvovirus B19 testing which revealed positive IgM titers. Symptomatic treatment was provided and the patient was well in 6 weeks. ANA remained positive for 6 months.

#### Concluding diagnosis

Virally induced ANA and autoimmune syndrome due to Parvovirus B19.

#### Take home points

Human Parvovirus B-19 infection is common during childhood and can rarely mimic SLE in children as in this case. Clinical and laboratory features are similar, including ANA positivity (32). ANA positivity and illnesses mimicking pSLE are associated with other viral infections, including EBV, hepatitis C (HCV) (18, 31), and recently SARS-CoV-2 (60, 61). While environmental factors are thought to trigger SLE in genetically susceptible individuals, there is no clear evidence that viruses cause pSLE (32). Moore et al. demonstrated that children with Parvovirus B19 may also transiently express antibodies to specific nuclear antigens suggesting a possible mechanism for the generation of autoantibodies (32). It is suggested that immunogenic viruses like EBV and Parvovirus could be the trigger that tips a genetically predisposed child to develop pSLE.

Another common virus affecting populations world-wide is hepatitis C (HCV). HCV is associated with positive ANA by IFA in up to 40% of chronic HCV patients (31). HCV infection is uncommon in children in many parts of the world, however, given the frequency of associated rheumatologic symptoms such as arthralgias and fatigue, risk factors for HCV must be reviewed when evaluating such symptoms in children. Some researchers propose that a positive ANA alters the disease course and outcome whereas other authors believe that ANA positivity is an immunological epiphenomenon with no bearing on pathogenesis or response to treatment (31).

### Case 6

#### Non-rheumatic disease positive ANA in children

A 12 year old has complaints of knee pain after soccer practice for the past 3 sports seasons. There is no joint swelling or stiffness reported and she has no other complaints. She is otherwise well. Due to the persistent symptoms, her primary care clinician obtains laboratory tests which reveal a normal CBC, normal ESR of 5 mm/h, normal biochemical profile, and a positive ANA by IFA of 1:160 in a DFS pattern. Radiographs of the knees were normal. There is a family history of osteoarthritis and asthma but no autoimmune disease or orthopedic disorders. The patient was referred to pediatric rheumatology. Her examination was normal except for patellar laxity with no inflammatory arthritis identified. The positive ANA was further evaluated with a few additional lab tests: TSH, Free T4 and anti-thyroid antibodies were negative. Patient and family education and reassurance were provided. Symptomatic treatment including an at-home physical therapy program was successfully implemented.

#### Concluding diagnosis

Patellar laxity and no autoimmune or inflammatory disease. The ANA is positive but not indicative of any specific diagnosis.

#### Take home points

This case exemplifies common patient referrals to pediatric rheumatology practice - undiagnosed but long-standing mechanical joint pain with no signs or symptoms to suggest an inflammatory condition (40, 62). The ANA test was performed not to diagnose pSLE nor to stratify risk of uveitis in JIA, but to investigate the possibility that the symptoms are due to autoimmune process (low pre-test probability of disease). A low titer ANA in this clinical scenario is a non-diagnostic positive result (1, 2, 16). Five to 30% of teens will have a low titer positive ANA in this scenario. The DFS70 ANA IFA pattern does not consistently correlate with specific diagnoses in children or adults and its presence in the setting of an isolated positive ANA may support the lack of an autoimmune diagnosis (48–50). Education and reassurance of the patient and family are required to clarify the lack of significance of this result. It is noteworthy that the ICD-10 provides a series of “ANA positive” codes for practitioners to use given this common diagnosis following consultation (6).

A positive ANA result of >1:80 titer does not usually predict development of autoimmune disease based on several long-term follow-up studies that tracked such patients over time. In many children, the ANA eventually became negative, and even in the 20% with persistently positive serology, no inflammatory autoimmune disorder developed (47, 57). Deane et al. found that only 1 of 31 patients with a positive ANA and no evidence of autoimmunity at initial evaluation did develop a non-rheumatologic autoimmune disorder, indicating a very low rate of progression (2). These reports further support that the ANA should not be tested with low pre-test probability of autoimmune disease.

## Discussion

Ordering and interpreting ANAs in children requires understanding of laboratory techniques, implications of positive results in the clinical scenario under investigation, and understanding the result report. Acquiring this knowledge is the best approach for the practicing clinician to avoid over-ordering and over-interpreting the ANA result.

Multiplex immunoassays have led to a paradigm shift in ANA testing for autoimmune diseases (63). Rapid throughput techniques have replaced traditional methods like IFA and ELISA in many clinical laboratories. However, this change has not improved reliability and reproducibility in ANA testing or ordering practices. To improve upon this issue in adults, Patel et al. developed an educational and clinical decision support approach to improve the reliability and PPV for adult rheumatology ANA referrals (64). Algorithms have been proposed previously but it does not appear that such stepwise approaches have been used consistently or the over-referral for “positive ANA” would have declined.

In SLE, the correlation between IFA, ELISA and multiplex assays is high in multiple studies, but in other diseases, such as JIA, there is poor correlation between assays. It is worrisome that widespread use of multiplex assays will miss virtually 100% of the JIA related ANA positivity (Case 1), putting these young children at higher risk of complications due to unrecognized uveitis. Ideally, identification of new serologic biomarkers to close the “seronegative gap” (43) and develop tests that detect disease-specific antigens in pSLE, JIA and AH will better classify, diagnose, and prognosticate in pediatric patients. Development of new methods using machine learning and artificial intelligence techniques to identify subtle patterns and correlations will further enable rheumatologists to care for their patients (43).

Reduction of unnecessary ANA ordering is key. Mohammed et al. found that the major reason for unnecessary ANA ordering appears to be lack of provider awareness regarding test characteristics and interpretation. The test names such as “ANA Direct” and “ANA Screen” are confusing misnomers as these tests are not actually measuring the ANA itself. When unexpected results are reported (Case 3), there is uncertainty in interpreting the test report often precipitating referral for rheumatology consultation plus additional testing (63). ANA determination using a two-step sequential process would be recommended for patients undergoing evaluation for SLE (Case 2). IFA would be performed first followed by multiplex or other assays with disease-specific antigens (59, 65). Hence, the ideal test of choice for possible SLE patients could be “ANA with reflex to multiplex” if available from commercial or institutional laboratories.

The ACR has collaborated with the AAP as part of the national, multispecialty “Choosing Wisely” campaign aimed at improving value-based testing and care (7). Pertinent to this discussion is the advice from the ACR and AAP to only order an ANA with strong suspicion of a rheumatic disease. In the campaign publication it states “The ANA has high sensitivity for only one disease, SLE, but has very poor specificity for SLE and every other rheumatic disease. Therefore, it is not useful or indicated as a general screen of autoimmunity. … Limiting patients on which to order ANAs would reduce unnecessary physician visits and laboratory expenses as well as parental anxiety. “Lupus panels” and other similar panels should also not be ordered without concerns for specific autoimmune disease. Additionally, since the ANA may always be positive and may fluctuate in titer, it is not recommended to retest it unless there is some new clinical concern.”

The ACR recommends ANA by IFA as the “gold standard” of testing when compared to solid phase multiplex assays which, despite the efficiency this platform brings, may fail to detect ANAs in as much as 32% of IFA positive children (9, 44) and up to 100% of JIA patients.

Key take-home points are:

1. Ordering providers should limit ANA testing to specific clinical contexts. For example: to rule out pSLE, to assess a JIA patient for risk of uveitis (IFA only), to determine whether a child with RP is at risk of a systemic autoimmune diseases (IFA only), and to diagnose AH (IFA only) or MCTD.
2. The IFA ANA testing using Hep-2 cells should remain the gold standard for ANA testing in children to avoid false negative ELISA and/or multiplex results.
3. Do not order multiplex testing for ANA determination in children to assess for possible rheumatic diseases.
4. Hospital and commercial laboratories using bead-based multiplex platforms or other solid phase assays for detecting ANAs must inform ordering providers about assay features and sensitivity and specificity compared to IFA assays.
5. Assays for detecting ANA as well as anti-DNA, anti-Smith, anti-RNP, anti-Ro/SS-A, anti-La/SSB, etc. should be held to national and/or international standards.
6. Laboratories should specify the methods used for measuring ANAs in result reports.

## Conclusions

The literature on ANA testing in children has been reviewed. Cases that exemplify typical patients referred to pediatric rheumatology were presented to demonstrate reliable and reproducible use of ANA testing in practice. Improved ANA utilization will benefit patients not only through timely and accurate diagnoses but also by limiting healthcare expenditures from unnecessary consultations and follow up testing. The IFA ANA test should remain the gold standard for ANA testing in children.

## Author contributions

The author confirms being the sole contributor of this work and has approved it for publication.
